# The cancer-testis antigen a-kinase anchor protein 3 facilitates breast cancer progression via activation of the PTEN/PI3K/AKT/mTOR signaling

**DOI:** 10.1080/21655979.2022.2051687

**Published:** 2022-03-24

**Authors:** Chuan-Hua Zhan, Dong-Shen Ding, Wei Zhang, Hong-Liang Wang, Zhe-Yu Mao, Guo-Jun Liu

**Affiliations:** a Department of Clinical Laboratory, Huangshi Central Hospital, Affiliated Hospital of Hubei Polytechnic University, Edong Healthcare Group, Huangshi, P.R. China; b Hubei Key Laboratory of Kidney Disease Pathogenesis and Intervention, Huangshi, P.R. China; c Department of Medical Oncology, Huangshi Central Hospital, Affiliated Hospital of Hubei Polytechnic University, Edong Healthcare Group, Huangshi, P.R. China; d Department of Breast Cancer Surgery, Huangshi Central Hospital, Affiliated Hospital of Hubei Polytechnic University, Edong Healthcare Group, Huangshi, P.R. China

**Keywords:** Cancer-testis antigen, A-kinase anchor protein 3 protein, breast neoplasms, prognosis, mechanism

## Abstract

The cancer-testis antigen A-kinase anchor protein 3 (AKAP3) has been shown to have a strong association with breast cancer (BC). However, its role in BC progression received scant attention. We aimed to explore the prognostic implication of aberrant AKAP3 expression for a better knowledge of BC progression and improved treatment. AKAP3 expression was quantitated using tissue microarrays and immunohistochemistry (IHC). Cell viability, invasion, migration, apoptosis, and expressions of PTEN/PI3K/AKT/mTOR signaling components were assessed in AKAP3-overexpressed or si-AKAP3-transfected BC cells. Finally, elevated AKAP3 expression was observed in BC versus paracancerous tissues. BC patients with high AKAP3 expression showed a worse prognosis than low expression patients (*P* < 0.0001). AKAP3 overexpressions fueled cell growth, proliferation, migration, and invasion in HCC1937 and MDA-MB-468 BC cell lines, alongside increased expressions of PI3K/AKT/mTOR signaling components and PTEN suppression. These effects were pronouncedly reversed, together with elevated apoptosis, in cells transfected with si-AKAP3. Therefore, AKAP3 is upregulated in BC and promotes BC cell growth, invasion, and migration via PTEN/PI3K/AKT/mTOR signaling activation. It may serve as a prognosis indicator for BC survival.

## GRAPHICAL ABSTRACT

**Figure:**
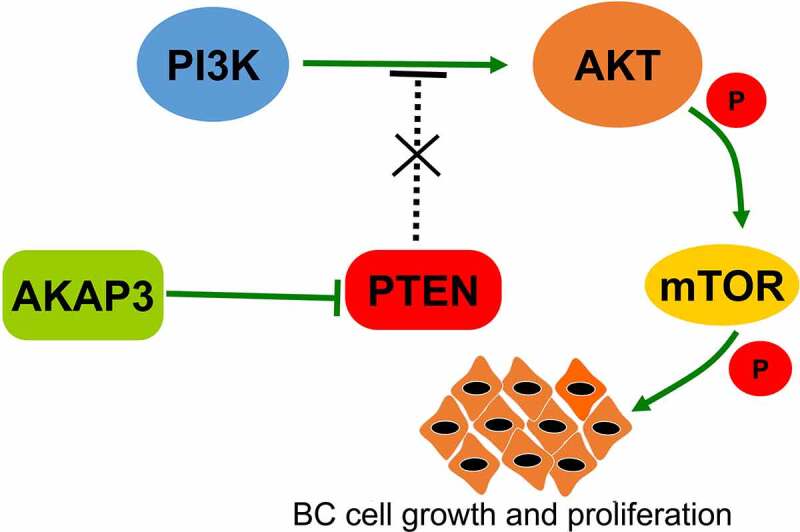

## Introduction

Breast cancer (BC) is a malignant tumor derived from the glandular epithelium of the breast. It shows region-specific prevalence in North America, northern Europe, and Oceania [1] and is the leading malignant tumor among women worldwide, with the rate of new cases growing at a higher-than-average speed of 3% per year [2]. It is also the fourth leading cause of death from cancer in China, according to the 2015 cancer statistics [3]. Except for known tumorigenic factors, such as serum hormone, unhealthy diet, a history of familial BC, and environment [2,4], proto-oncogenes, inactivation of tumor suppressor genes, mutations, and other genetic abnormalities also contribute to the occurrence of BC [5,6]. Currently, significant progress has been made in the identification of more biomarkers [7], thanks to the known biomarkers of BC survival or treatment and the application of new technologies in BC molecular research [8], which underlies robust diagnostic algorithms based on biomarker combinations. In this respect, comprehensive expression profile analysis of genes for identification of new biomarkers is still a feasible way to improve treatment decision-making and patient prognosis.

Cancer-testis antigens (CTAs) are frequently detectable in normal testicular tissues, spermatozoa, and various types of tumor cells in humans and other mammals and lowly expressed in normal somatic cells [9]. Current knowledge on CTA genes reveals that the majority of these genes belong to distinct multigene families and are located on the X chromosome [10]. They are primarily expressed in germ cells of the normal testis (instead of other normal tissues) and most malignant tumor cells derived from different types of tissues and elicit immune responses to antigenic stimuli in tumors [11]. Since the melanoma antigen gene (MAGE) family was discovered by Vander Bruggen in 1991 using T cell epitope cloning [12], more than 200 CTA genes have been identified and shown to have close associations with mutations in tumorigenic genes or tumor suppressor genes during gametogenesis [13]. Therefore, CTAs are considered as one of the most promising targets for cancer vaccine therapy, of which MAGE-A, IL-13R*α*, and NYESO-1 have been studied in patients to verify whether they can cause tumor regressions [9,14,15].

Human A-kinase anchoring protein 3 (AKAP3) is a CTA family member essential for tumorigenesis. The human *AKAP3* gene that maps on chr12:4,615,508–4,649,047 and encodes AKAP3 is a member of the AKAP family of functionally related proteins that target protein kinase A (PKA) to discrete locations within the cell. It has been shown to interact with the R-subunit of PKA and sperm-associated proteins to regulate motility, capacitation, and the acrosome reaction [16]. Its roles in the occurrence and development of ovarian cancer have been reported [17]. However, there are limited studies on AKAP3’s roles in distinct tumors.

The PI3K/AKT/mTOR signaling has been shown to involve in cell differentiation, proliferation, anti-apoptosis in breast cancer cells, and oxidative stress and angiogenesis [18]. PETN is an inhibitor of the PI3K/AKT/mTOR signaling. PTEN methylation, alongside PI3K mutations and AKT activation, has been reported to be closely associated with response and resistance to anticancer therapy, particularly hormonal therapy resistance, and prognosis in BC [19]. Currently, direct evidence of the correlation between AKAP3 and the PI3K/AKT/mTOR signaling is unavailable, but CTA research in lung cancers has demonstrated the relationship of another cancer-testis antigen LDHC with this signaling, which may promote GSK-3*β*-dependent phosphorylation of AKT [20]. AKAP3 is a dual-specificity molecule that modulates PKA isotypes. The latter can trigger the PI3K/AKT signaling in MCF-7 breast cancer cells [21,22]. Thus, it is intriguing to hypothesize that AKAP3 restoration or downregulation may alter the activity of the PI3K/AKT/mTOR signaling. In the present study, we aimed to characterize the expression pattern of AKAP3 in BC using high-throughput tissue microarrays and IHC and explore correlations between aberrant AKAP3 expression and clinicopathological features and prognosis of BC patients. We used transfections to block or overexpress AKAP3 in human BC cell lines. We hypothesized that AKAP3 could promote BC malignant behavior via PI3K/AKT/mTOR signaling activation.

## Materials and methods

### Clinical data

*AKAP3* gene expression in BC and paracancerous tissues was quantitated using a tissue microarray (HBreD145Su02, SHANGHAI OUTDO BIOTECH) containing BC tissue samples from 145 female patients (median age: 60 years, range: 31 to 88 years) who were diagnosed with BC at our center between August 2004 and December 2008 and a paracancerous tissue microarray (HBreDuc090Sur01, SHANGHAI OUTDO BIOTECH) comprising paracancerous tissue samples from 90 patients enrolled between January 2001 and June 2004. BC pathological subtypes included invasive ductal carcinoma or lobular carcinoma (142 cases), mucinous carcinoma (2 cases), and intraductal carcinoma (1 case). Two of them were triple-negative breast cancer (TNBC) cases. All cases were confirmed to have primary cancer without distant organ metastasis. Sixty-nine cases had lymph node metastasis, and 70 showed the absence of lymph node metastasis. In six cases, the presence of metastasis was not recorded. The sixth edition of AJCC Staging System was utilized to stage BC patients as follows: stage 0 (*n* = 1), stage 1 (*n* = 22), stage 2 (*n* = 78), and stage 3 (*n* = 39). Five participants had missing stage records (*n* = 5). A tissue microarray (HBreD050Bc01, SHANGHAI OUTDO BIOTECH) comprises 24 TNBC cases were also used for the analysis. All patients were followed up until July 2014. Approval for tissue microarray analysis was given by the research ethics committee.

### Immunohistochemistry (IHC)

AKAP3 expression in primary BC tissues (*n* = 169) was determined using an EliVisionTM Plus IHC Kit (MXB Biotechnology, China). Tissue blocks were dewaxed in xylene and dehydrated through a graded ethanol series. Samples were treated with hydrogen peroxide (H_2_O_2_) for 10 min. Antigen retrieval was performed by boiling sections for 3 min in the citric acid buffer in a pressure cooker (100–120°C). Sections were incubated with rabbit polyclonal anti-human AKAP3-antibody (Proteintech) diluted 1:50 for 90 min at 37°C, washed in PBS, and incubated with a goat anti-rabbit secondary antibody for 30 min at room temperature. Samples were visualized by incubation with diaminobenzidine (DAB) substrate buffer for 2 min and counterstained with hematoxylin. All sections were reviewed by two experienced pathologists blinded to samples and the patients’ clinical data. Immunoreactivity was scored based on color intensity: unstained (0 points), light brown (1 point), brown (2 points), dark brown (3 points). The scoring system based on the proportion of AKAP3-positive cells was used: < 5% (0 points), 5%-25% (1 point), 25%-50% (2 points), > 50% (3 points). The AKAP3 expression score was calculated by multiplying the proportion score by color intensity score and graded accordingly: 0 (−), 1–2 (+), 3–5 (++), 6–9 (+++), of which −/+ was regarded as low expression and ++/+++ high expression [23].

### *Cell culture, vector construction, and construction of* AKAP3*-overexpressed and silenced BC cell lines*

Human MCF-7, HCC1937, MDA-MB-231, and MDA-MB-468 BC cell lines, and Hs578Bst breast cell line and HEK293a cell line (Shanghai Cell Bank of Chinese Academy of Sciences, Shanghai, China) were selectively cultured in DMEM high-glucose medium or RPMI-1640 medium containing 10% FBS in a 37°C, 5% CO_2_ incubator. MDA-MB-231 cells were maintained in Leibovitz’s L-15 medium supplemented with 10% FBS in a CO_2_-free incubator at 37°C. The culture medium was replaced with a fresh medium every 2–3 days, and cells were passaged every 5–7 days. GV358-EGFP lentivirus vectors overexpressing AKAP3 (Oe-AKAP3) and GV115-EGFP vectors expressing RNA interference (RNAi) against AKAP3 (si-AKAP3) were successfully constructed and packaged in cells. AKAP3-overexpressed or silenced cell lines were screened using 0.4 μg/mL puromycin.

### Real-time qRT-PCR

Total RNA was extracted from cell lysates using a cell/tissue RNA extraction kit (Promega) and reversely transcribed into cDNA using the Transcriptor First-strand cDNA Synthesis Kit (ROX). The cDNA was amplified using the ABI7500 fluorescence quantitative PCR system and SYBR Green Master (ROX) with *AKAP3*-specific forward (5’-ACAAGGCTGAGAGTTATTCCCT-3’) and reverse primers (5’- CTCACCCAGAGTTTTCGCACA-3’, product length: 158 bp). The primers of the housekeeping gene *GAPDH* included forward 5’- GACAGTCAGCCGCATCTTCT-3’ and reverse 5’-GCGCCCAATACGACCAAATC-3’ (product length: 104bp). The PCR cycling conditions were as follows: one cycle of pre-denaturation at 95°C for 10 min, 40 cycles of denaturation at 95°C for 15s, and one cycle of annealing at 60°C for 1 min. Relative *AKAP3* mRNA expression was calculated as a fold change of 2^−ΔΔCT^ value. The Hs578Bst breast cell line as normal control, and HEK293a cell were treated as AKAP3-negative control.

### Cell viability assay

Cells in the logarithmic growth phase were adjusted to 5 × 10^3^ cells/well and cell suspension was prepared with DMEM high-glucose medium or RPMI-1640 medium. 100 μL per well was inoculated in a 96-well culture plate. After culture for 0, 24, 48, 72, and 96 h, 10 μL of CCK-8 reagent was added to each well and cultured for 3 h. The optical density (OD) values were measured at dual wavelengths (450 and 630 nm) using a microplate reader (Thermo), which was proportional to the number of cells. The percent viability of each sample was calculated depending on the OD values of four replicate wells. Growth curve analysis was performed to monitor cell proliferation [24].

### Wound healing migration assay

BC cells (5 to 8 × 10^5^ cells/well) were seeded into a 6-well plate and incubated to a confluence of 95% or more. A vertical scratch wound was made by scraping a line down the center of each well with a disposable 200-μL micropipette tip. Nonadherent cells were removed with two washes of PBS, and remnant cells were photographed at 0, 48, and 72 h. The number of migrated cells was counted and compared to baseline values (0 h) to reflect cell migration ability [25].

### Transwell invasion and migration assay

Cell concentration was adjusted to 1.5 × 10^5^ cells/mL. To perform a Transwell assay, Transwell chambers containing Matrigel-coated filters with an 8-µm pore size were utilized. Matrigel-coated filters were not employed in migration assays. BC cell suspension (200 μL per well) was added to the upper chamber, and 600 μl of culture medium containing 30% fetal bovine serum was added to the lower chamber. Cells were allowed to invade or migrate for 48 or 72 h. The cells in the lower chamber were fixed with methanol for 30 min and stained with 0.1% crystal violet dye solution for 20 min. After washes with PBS, cells were photographed and counted under a microscope. Five fields were randomly selected, and the number of cells migrating across a filter was counted to assess cell invasion or migration ability [25].

### TUNEL assay

MCF-7 and MDA-MB-231 cells were collected, washed, and fixed with 4% paraformaldehyde for 30 min and then incubated with PBS containing 0.3% Triton X-100 for 5 min at room temperature. Cells were incubated in 0.3% H_2_O_2_ in PBS for 20 min at room temperature to suppress endogenous peroxidase activity. Subsequently, a biotin-conjugated reagent (50 μL/well) was added to each well for 60 min at 37°C. After one wash with PBS, cells were incubated with stop solution (0.1–0.3 mL/well) for 10 min at room temperature and with 50 μL of streptavidin solution (Beyotime, China) for 30 min at room temperature. The reaction was visualized with DAB (0.2–0.5 mL/well). The nucleus was counterstained with hematoxylin. Cells were assessed as apoptotic when the nucleus was stained brown or dark brown. For each group, apoptotic cells were counted in five fields randomly selected and the data were analyzed [26].

### DNA ladder assay

DNA laddering is a significant indicator of cell apoptosis. Cell apoptosis can be confirmed while observing DNA ladder formation. Therefore, DNA ladder assays were performed. Trypsin-digested cells were washed once with PBS or saline and centrifuged at 1,000–2,000 × g for 1–2 min. Cell lysates were mixed with protease K (5 μL/1 mL), vortexed, and placed in a tube in the water bath at 50°C overnight to digest. Tris-equilibrated phenol solution (500 μL, pH 8.0) was added to the cell lysate and thoroughly vortexed. The supernatant was extracted, mixed with 60 μL of 10 M ammonium acetate and 600 μL of anhydrous ethanol, and turned upside down several times until DNA precipitation occurred. The mixture was centrifuged at 12,000 × g for 10 min at 4°C, and the supernatant was removed. DNAs were washed once with 70% ethanol, dried, and dissolved in TE buffer. DNA fragmentation was measured using 1% agarose gel electrophoresis [27].

### Immunoblotting

Immunoblotting was conducted following the procedures by the published study [28]. Briefly, cells were collected by centrifugation, and total protein was extracted with RIPA buffer. Proteins were separated by 5%-12% SDS-PAGE gels (80–120 V), transferred to the PVDF membrane at 180 V, and blocked in 5% skimmed milk in TBST overnight. The membranes were incubated with the following primary antibodies (mouse or rabbit anti-human) for 4 h at room temperature: anti-human AKAP3 (1:500; Invitrogen, PA5-109,376), anti-PTEN (1:500; Beyotime, AP686), anti-PI3K (1:500; Abcam, ab154598), anti-AKT (1:500; Abcam, EPR16798), anti-mTOR (1:500), anti-pAKT (phospho T308) (1:200; Abcam, ab38449), anti-pmTOR (phospho S2448) (1:200; Abcam, ab131538), and anti-*β*-actin (1:1000; Beyotime, AF0003) antibodies. After three washes with TBST, membranes were incubated with Horseradish peroxidase (HRP)-conjugated goat anti-mouse/rabbit IgG secondary antibody (1:5000) for 2 h at room temperature. Protein bands were visualized using the ECL luminescent solution, photographed, and quantitated using the Image J software.

### Statistical analysis

SPSS 20.0 software (IBM Corp., Armonk, NY, USA) was used for all statistical analyses. Measurement data were presented as the mean ± standard deviation (SD). The two-sample *t*-test was applied for normally distributed continuous variables. Differences in categorical variables (e.g., clinicopathological features and AKAP3 expression scores) between groups were compared using the *Chi*-square test. Kaplan-Meier survival analysis was performed to assess potential correlations between aberrant AKAP3 expression and overall survival (OS). A *P*-value of *<* 0.05 was considered statistically significant.

## Results

### AKAP3 serves as a prognostic marker in BC

**Figure 1.**
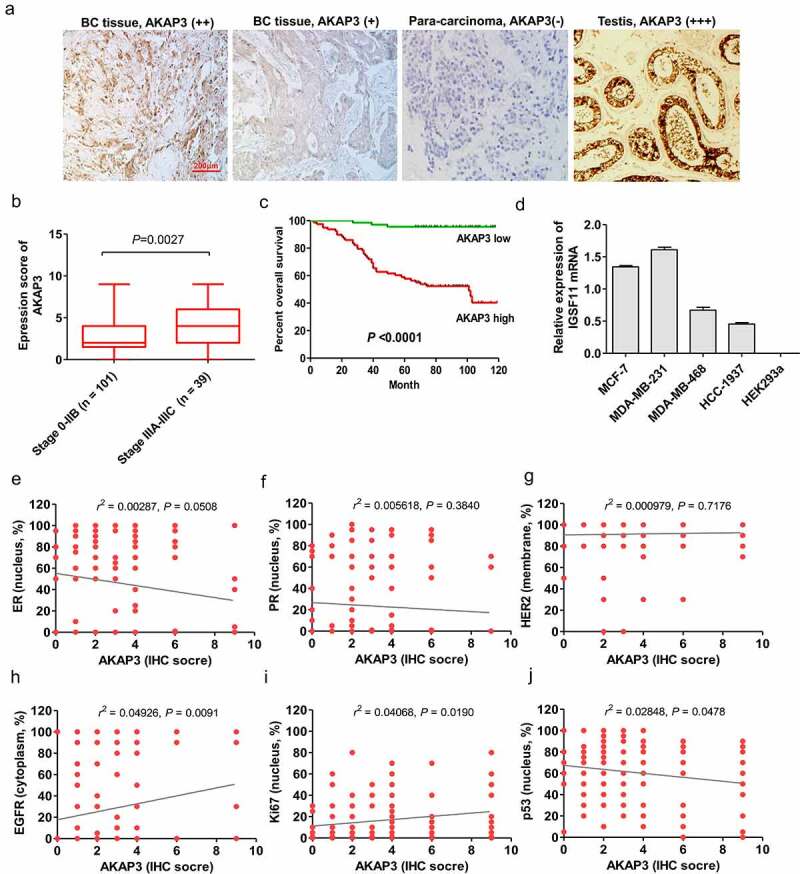
Expression of AKAP3 in BC and correlation of AKAP3 expression with prognosis. (a) IHC analysis shows AKAP3 upregulation in BC versus testicular tissues (positive controls) (200× magnification). (b) Two-tailed t test showed a higher AKAP3 expression score in stage 3A-3C versus 0-IIB BC patients . (c) BC patients with AKAP3 low expression patterns can enjoy longer overall survival (OS) than those dominating higher expression patterns. (d) AKAP3 expressions in HEK293a cell lines. Correlations of AKAP3 expression with (e) ER, (f) PR, (g) HER2, (h) EGFR, (i)Ki67, and (j) p53.

AKAP3 expression patterns in BC tissue and cell lines and the prognostic significance of aberrant AKAP3 expression were assessed. AKAP3 was primarily expressed in the cytoplasm of tumor cells (Figure 1(a)) and was detectable in 136/145 (93.8%) BC tissue samples, including 79 cases with AKAP3 high expression patterns (++/+++) and 57 with AKAP3 low expression patterns (±/+). Moreover, the positive expression rate of AKAP3 was estimated to be of 65.38% (17/26) in TNBC cases. In paratumorous tissues (*n* = 90), AKAP3 was lowly expressed (±/+) in 28 cases and undetectable (−) in 62 cases. AKAP3 expression IHC score significantly increased in stage 3A-3C versus 0–2B BC patients (*P* = 0.0027, Figure 1(b)). However, there were no significant correlations of AKAP3 upregulation with clinicopathological prognostic parameters (Table 1). BC patients with low AKAP3 expression could enjoy longer OS than those with high AKAP3 (Figure 1(c)) (*P < *0.0001). Similarly, *AKAP3* mRNA expression was significantly elevated in MDA-MB-231 and MCF-7 BC cells compared to that achieved in MDA-MB-468 and HCC1937 cells. HEK293a cell line failed to express *AKAP3* mRNA and was regarded as negative control (Figure 1(d)). Further, AKAP3 expression had a positive correlation with EGFR and Ki67 expressions, a negative correlation with p53, but no associations with HER2, ER and PR (Figure 1(e-j)).

**Table 1.** Correlations between AKAP3 expression and clinicopathological features in BC patients

Clinicopathological parameters	*n*	AKAP3 Low (*n*)	AKAP3 high (*n*)	*P* value *(X^2^ test)*	Loss of information (*n*)
Age (years)				0.448	0
>50	106	51	55		
≤50	39	16	23		
Clinical stage				0.368	5
0-II	101	50	51		
III	39	16	23		
Tumor size				0.222	4
>2 cm	103	45	58		
≤2c m	38	21	17		
HER2 expression				0.181	6
Positive	41	15	26		
Negative	98	48	50

### AKAP3 overexpression promotes BC cell proliferation, invasion, and migration

**Figure 2.**
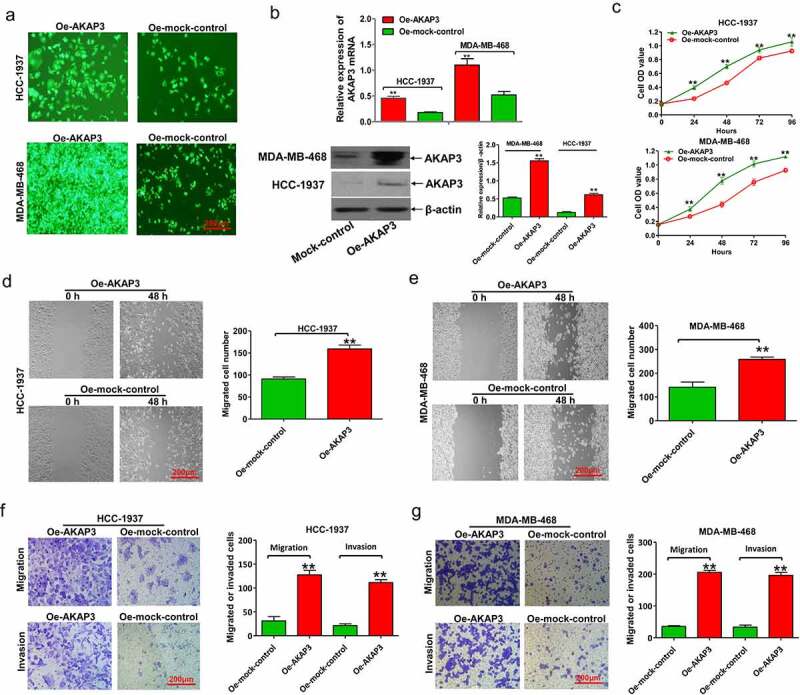
AKAP3 overexpression promotes cell growth, invasion, and migration in HCC1937 and MDA-MB-468 cell lines. (a) The EGFP assay reveals that AKAP3 is highly expressed in HCC1937 and MDA-MB-468 cell lines transfected with Oe-AKAP3. (b) AKAP3 mRNA and protein expressions significantly increase in HCC1937 and MDA-MB-468 cell lines transfected with Oe-AKAP3. (c) The CCK-8 assay shows the growth curve of BC cells. Growth is measured as OD values at 450 and 630 nm. The OD value is proportional to the number of cells. (d, e) The wound-healing assay shows increased cell migration in AKAP3-overexpressing cell lines at 48 h. (f, g) Transwell assay reveals boosted cell invasion ability in AKAP3-overexpressing cells at 48 h. *P < 0.05 and ** *P* < 0.01 vs. mock control.

To confirm whether AKAP3 overexpression could affect cell viability, we performed CCK-8, wound healing migration, and Transwell invasion and migration assays in HCC1937 and MDA-MB-468 cell lines whose AKAP3 was endogenously downregulated. HCC1937 and MDA-MB-468 cells overexpressed AKAP3 at mRNA and protein levels after transfection with Oe-AKAP3 (Figure 2(a,b)). Compared to sh-NC, AKAP3-overexpressing cells had greater proliferation (Figure 2(c)), migration, and invasion abilities in both cell lines (Figure 2(d-g)).

### AKAP3 knockdown suppresses BC cell growth, proliferation, invasion, and migration

**Figure 3.**
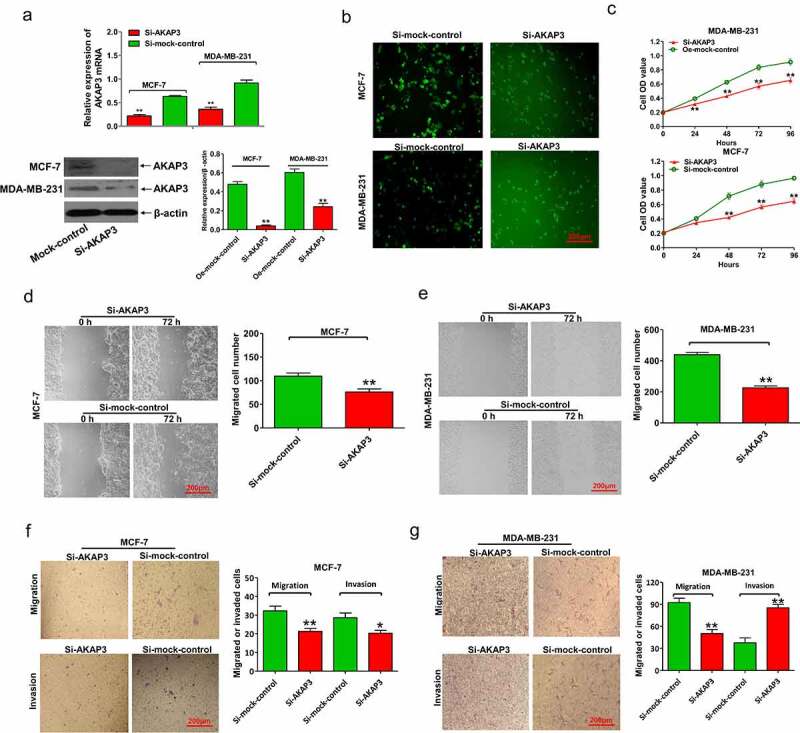
AKAP3 knockdown inhibits BC cell growth, proliferation, invasion, and migration. (a) AKAP3 mRNA and protein expressions are significantly decreased in si-AKAP3-transfected MCF-7 and MDA-MB-231 cell lines. (b) A lower number of BC cells after si-AKAP3 transfection. (c) The CCK-8 assay shows inhibited vitality of BC cells. (d, e) The wound-healing assay reveals reduced cell migration in si-AKAP3-transfected cells at 72 h. (f, g) Transwell assays show suppressed invasion ability in si-AKAP3-transfected cells at 72 h. *P < 0.05 and ** *P* < 0.01 vs. mock control.

We chose MCF-7 and MDA-MB-231 cell lines that had endogenous high AKAP3 for knockdown experiments. In qRT-PCR and immunoblotting assays, AKAP3 mRNA and protein levels sharply decreased in si-AKAP3-transfected MCF-7 and MDA-MB-231 cells (Figure 3(a)). As shown in Figure 3(b), the number of MCF-7 and MDA-MB-231 cells pronouncedly decreased after si-AKAP3 transfection, together with suppressed cell growth and proliferation (Figure 3(c)), compared to empty vector (sh-NC) transfected cells. In Transwell invasion and migration assays, si-AKAP3-transfected cells exhibited suppressed invasion and migration abilities compared to sh-NC cells (Figure 3(d-g)). These findings suggest that AKAP3 knockdown inhibits the malignant behavior of MCF-7 and MDA-MB-231 cells.

### AKAP3 knockdown triggers BC cell apoptosis

**Figure 4.**
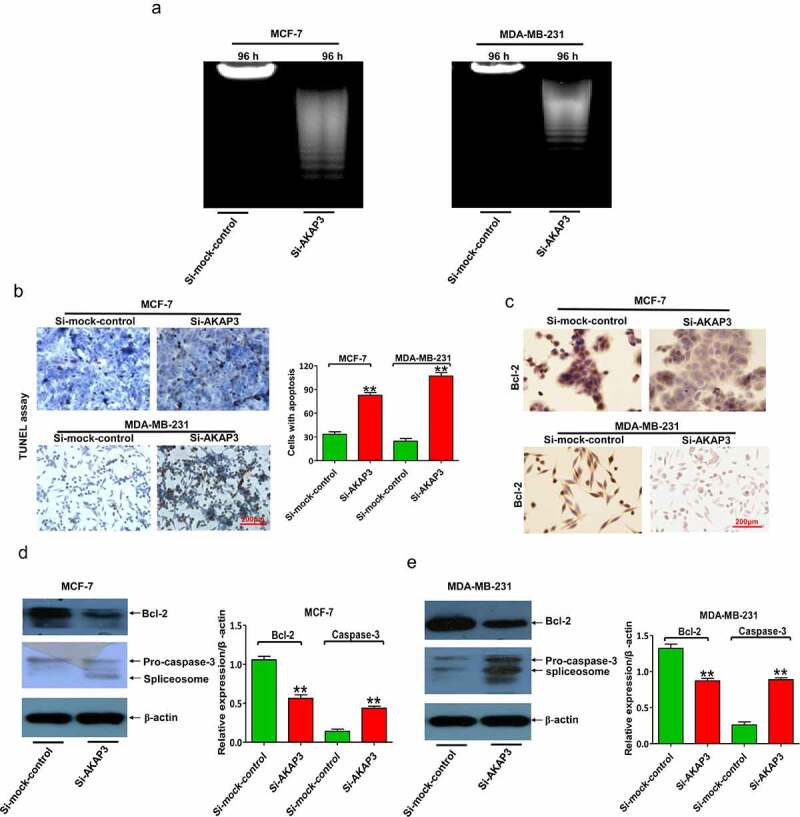
AKAP3 knockdown induces cell apoptosis in MDA-MB-231 and MCF-7 cells. (a) The DNA ladder assay shows enhanced nuclear DNA degradation in apoptotic BC cells and a drastic decrease in total DNA at 96 h. (b) TUNEL assay shows fewer apoptotic cells (brown) in si-AKAP3-transfected cells versus empty vector (sh-NC) transfected cells (200× magnification). (c) IHC assay reveals Bcl-2 protein expression in si-AKAP3-transfected versus sh-NC cells (200× magnification). (d, e) Immunoblotting assay reveals lower Bcl-2 expression and higher caspase-3 expression in si-AKAP3-transfected versus sh-NC cells. *P < 0.05 and ** *P* < 0.01 vs. mock control.

Apoptosis analyses were conducted to investigate whether the suppressive effects of AKAP3 downregulation on cell viability were due to apoptosis. MCF-7 and MDA-MB-231 cell lines were selected. We found that compared to sh-NC nuclear DNA degradation was enhanced in apoptotic cells, together with a more drastic decrease in total DNA at 96 h, after AKAP3 knockdown (Figure 4(a)). An increase in the number of apoptotic cells was detectable in the si-AKAP3 group (Figure 4(b,c)), which was consistent with downregulated anti-apoptotic Bcl-2 and elevated proapoptotic caspase-3 in transfected cells (Figure 4(d,e)).

### AKAP3 promotes the malignant behavior of BC cells via activation of PTEN/PI3K/AKT/mTOR signaling

**Figure 5.**
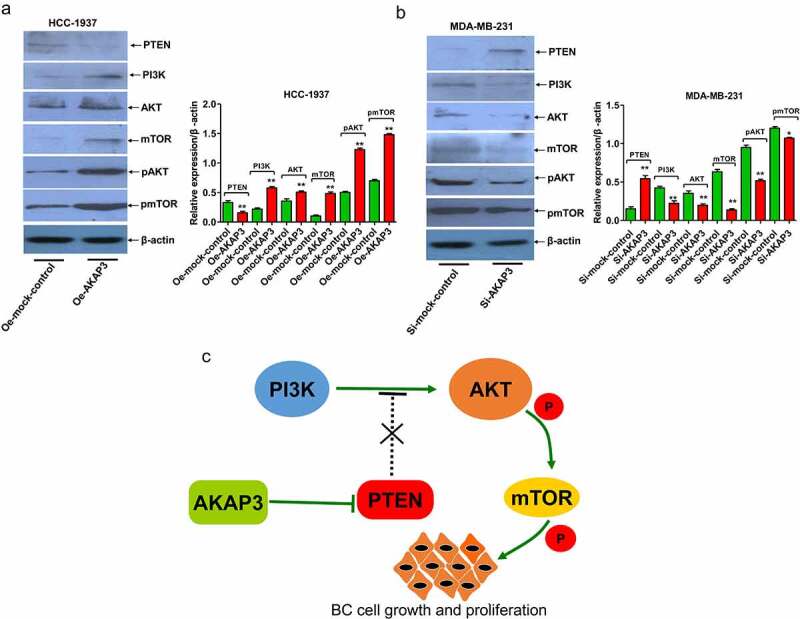
Effects of AKAP3 overexpression and knockdown on the expressions of PTEN/PI3K/AKT/mTOR signaling components. (a, b) expressions of PTEN, PI3K, AKT/pAKT, and mTOR/pmTOR in Oe-AKAP3-transfected HCC1937 cells and si-AKAP3-transfected MDA-MB-231 cells. (c) The mechanism responsible for AKAP3-mediated PTEN/PI3K/AKT/mTOR signaling. *P < 0.05 and ** *P* < 0.01 vs. mock control.

We selected AKAP3-overexpressing HCC-1937 cell line and si-AKAP3-transfected MDA-MB-231 cell line to verify correlations of AKAP3 with expressions of PTEN/PI3K/AKT/mTOR signaling components. Compared to the NC group, PI3K, AKT/pAKT, and mTOR/pmTOR expressions significantly increased, and PTEN (an inhibitor of the PI3K/AKT/mTOR signaling) expression decreased in Oe-AKAP3-transfected HCC-1937 cells (Figure 5(a)). The complete opposite results were detected in si-AKAP3-transfected MDA-MB-231 cells (Figure 5(b)). AKAP3’s regulation of the PTEN/PI3K/AKT/mTOR signaling was summarized in Figure 5(c). These findings suggest that AKAP3 promotes the malignant behavior of BC cells via triggering the PTEN/PI3K/AKT/mTOR pathway.

## Discussion

Evaluation of biomarker state or endpoints remains a valid instrument designated to screen the target population and monitor the efficacy of BC treatment. Since the immunogenicity of BC cells was previously reported [29], CTA has been extensively explored and proven an effective biomarker for immunotherapy in BC [30,31]. Two ongoing clinical trials focus on immunotherapy with MAGE-A- and NY-ESO-1-based polyvalent vaccines in patients with primary or metastatic BC [32]. Better knowledge of mechanisms linking roles of CTA genes aids in the development of anti-CTA targeted immunotherapy for BC, particularly advanced BC. AKAP3 is a member of the CTA family and is specifically expressed in the testis of humans and other mammals [16, 17, 33]. The efficacy of AKAP3 as a prognostic biomarker and an immunotherapy target has been demonstrated in ovarian cancer [33] and hepatocellular carcinoma [34]. However, its efficacy in BC has not been reported. In the present study, we demonstrated AKAP3 upregulation in BC tissues using the high-throughput BC tissue microarray combined with IHC. The results showed that AKAP3 overexpression had strong correlations with stage 3A-3C and shorter OS of BC patients. AKAP3 overexpression significantly enhanced malignant behavior in four BC cell lines, which was robustly reversed by AKAP3 knockdown via activation of the PTEN/PI3K/AKT/mTOR signaling.

Few studies in the literature focus on AKAP3 expression patterns in BC. In this study, we for the first time reported the positive expression rate of AKAP3 in BC tissues, as high as 93.8%. Higher AKAP3 expressions (histo-score) were observed in advanced- (stage 3A-3C) versus early-stage [stage 0–2B) BC patients. Further, AKAP3 expression was positively correlated with EGFR and Ki67 expressions and inversely related to p53 expression. These findings indicate that AKAP3 upregulation participates in the occurrence and development of BC. AKAP3 upregulation also had a significant correlation with poor OS of BC patients, suggesting that AKAP3 may serve as a prognostic indicator for survival in BC. However, the AKAP3 expression pattern differs among BC subtypes. Esmaeili et al. reportedthat AKAP3 was downregulated in triple-negative BC (TNBC] and high AKAP3 expression had a significant association with a better 5-year disease-free survival [35]. However, our subtype analyses showed that a high AKAP3 expression rate (65.38%, 17/26) was observed TNBC patients. A possible reason for these inconsistent correlation findings can be prognostic curves based on *AKAP3* mRNA expression in Esmaeili’s study versus those based on AKAP3 protein expression in the present study. It was reported that the percentage agreement between gene and protein expression was only 40%-50%, which underlies the difference between prognosis analysis results based on gene and protein expression data [36]. Future studies systematical assessment of aberrant AKAP3 expression in each BC subtype are needed.

We observed significantly enhanced cell growth, proliferation, invasion, and migration in HCC1937 and MDA-MB-468 cells after Oe-AKAP3 transfection. These effects were robustly inhibited by si-AKAP3, alongside increased apoptosis, in MCF-7 and MDA-MB-231 cell lines. This oncogenic effect of AKAP3 has been reported in other cancer types. [37] demonstrated that *AKAP-3* was highly expressed in patients with epithelial ovarian cancer and confirmed its role as an oncogene in epithelial ovarian cancer.

The PI3K/AKT/mTOR signaling is critical in cell-cycle regulation, which is directly involved in cell dormancy, proliferation, carcinogenesis, and lifespan of BC cells [33,38]. PTEN selectively antagonizes the PI3K/AKT/mTOR pathway [39]. PI3K activation converts phosphatidylinositol 4,5-bisphosphate [PI(4,5)P2] to the second messenger PIP3. The latter binds to the PH domain of the intracellular signal transduction proteins AKT and PDK1, thus phosphorylating AKT at Ser308 via PDK1 activation [40]. Activated AKT regulates cell behavior by phosphorylating downstream enzymes, kinases, and transcription factors [40]. In this study, we found that AKAP3 overexpression downregulated PTEN expression and activated the PI3K/AKT/mTOR signaling, promoting cell growth, proliferation, invasion, and metastasis in BC cells. These effects were potently suppressed by AKAP3 knockdown. Our findings indicate that AKAP3 regulates the malignant behavior of BC cells via activation of the PTEN/PI3K/AKT/mTOR signaling.

## Conclusion

AKAP3 is upregulated in BC cancerous tissues. AKAP3 elevation triggers the malignant behavior of BC via PTEN/PI3K/AKT/mTOR activation. It is associated with poor survival of patients with advanced BC, suggesting the potential of high AKAP3 to serve as a biomarker for prognosis prediction or a potential target for the immunotherapy for BC. We plan to carry out transcriptome and proteomic analysis in cell lines stably transfected with the *AKAP3* gene in the future to screen regulators associated with AKAP3’s effects on BC and related mechanisms.
